# Natural Environment and Social Relationship in the Development of Attentional Network

**DOI:** 10.3389/fpsyg.2020.01345

**Published:** 2020-06-26

**Authors:** Francesca Federico

**Affiliations:** Department of Developmental and Social Psychology, Sapienza University of Rome, Rome, Italy

**Keywords:** natural environment, attentional network development, social attention, stress, social relationships

## Abstract

The attention mechanism is related to both voluntary and automatic processes, that may be summarized in three distinct networks: alert, orientation, and inhibitory control. These networks can be modulated by different contextual and relational situations. Aim of this review is to explain how a combination of natural and social stimuli can positively affect the attentional processes. It has been proposed that the exposition to natural environment can positively affect direct attention, a common resource supporting both executive functioning and self-regulation processes in cognition. It has been suggested that the decrease of the effort required to voluntary control attention from the bottom upwards could determine some internal reflection that may support creative thinking secondarily to a simultaneous reduction in the effort required to orient attention between thoughts and impressions. In my view, not only exposition to natural and green environment improves attentional processes but also the involvement in social relationship. The development of the orientation and inhibitory control networks is sensitive to the social nature of the stimuli, for instance, in a task, including socially relevant stimuli the efficiency of these two attentional networks increases in children, in adults and in elderly subjects. Social attention, starting very early in the life (joint attention) is a very important mechanism for the regulation of social relationships. A key for a better development of cognitive functions such as attentional processes is the promotion of the immersion in the natural environment and the involvement in social relationship.

## Development and Modulation of Attentional Processes

One of the most qualified theories on attention with solid experimental support (e.g., Posner and Rothbart, 2007) is the Attention Network Theory (Posner and Petersen, 1990) that considers the attention processes divided into three anatomically and functionally different networks: alert, orientation, and executive conflict. The alert system is an automatic process responsible for maintaining the activation state allowing the rapid identification of environmental unexpected stimuli; the orientation system is responsible of the voluntary direction of attention toward a stimulus of interest; finally, the executive conflict is involved in solving challenging actions in tasks where conflicts are presents. The alerting system has a high performance in the requiring attention tasks, it seems to be developed at an premature age since infants show an improved capability to preserve the alert state during the first year of life (Rueda et al., 2005). The orienting response seems to be related to a diffuse neural network, involving the frontal eye fields (Wardak et al., 2006), the superior parietal lobe and temporal-parietal junction (Fuentes and Campoy, 2008), the superior colliculus, and the pulvinar nucleus of the thalamus (Shipp, 2004).

These three distinct networks have a different development *t* and also their interaction changes from childhood to adulthood (Mullane et al., 2016).

For example, when the alerting system is assessed by matching reaction times to targets with and without visual warning cues, 5-year-old subjects exhibited a more evident reduced alerting effects than 7-year-old children (Mezzacappa, 2004); 10-year-old children showed better alerting effects than adults (Rueda et al., 2004a), suggesting that children have higher difficulties than adults in preserving an alert state over time (Curtindale et al., 2007) without any exogenous cues.

The improvement of the orienting network has been very much investigated by cognitive (e.g., Akhtar and Enns, 1989; Enns and Brodeur, 1989; Schul et al., 2003; Leclercq and Sieroff, 2013) and clinical researches (e.g., Huang-Pollock and Nigg, 2003; Alvarez and Freides, 2004). Orienting oneself toward the source of information is the first operation to be done and it is necessary before moving on to other cognitively more complex operations (Wainwright and Bryson, 2002).

In consequence, this ability should be earlier developed and it is present in a more simplified form since the age of 4–6 months (Colombo, 2001). Exogenous orienting, that is stable across all the lifespan, is well developed by the age of 6 years (Plude et al., 1994).

Executive attention, that seems to be present in a very simple way in 6–7 months old infants, (Berger et al., 2006; Sheese et al., 2008) is further developed during middle and late childhood (e.g., Band et al., 2000). Rueda et al. (2004a,b) found an evident improvement of executive attention between 3 and 7 years of age. Although much of this development is under genetic control, it is also likely that the home and school environment and some specific training can influence it, as reported for other cognitive networks (Shafritz et al., 2004; Rueda et al., 2005).

## The Different Contextual Environment Can Differentially Affect Attentional Processes: the Urban Environment Versus the Natural Environment

Attentional processes are on the basis of extraction from the environment several characteristics useful to target activities. Different contexts may elicit crucial different patterns of attention selection. Since this is an active cognitive process, the attentional process, and in particular voluntary attention, has a very high individual costs. The attention restoration theory (ART), proposed by Kaplan in 1989 and more recently receiving attention in many articles, hypothesized that natural contexts are able to renew attention after exerting mental energy. ART suggests that the natural context provides the opportunity to avoid everyday stresses, experiences distensible spaces and contexts (“extent”), take part in activities that are “compatible” with our intrinsic motivations, and seriously faces stimuli that are “softly fascinating” (Kaplan, 1995; Ohly et al., 2016). This arrangement of elements stimulate “involuntary” or “indirect attention” and allows our “voluntary” or “directed” attention space to repair and renew (Kaplan, 1995). A recent review by Stenfors et al. (2019), conducted on different samples of students, showed that the natural environment provided several cognitive benefits on executive cognitive tasks with high implication on directed attention processes. Cognitive performance significantly improves after to be immersed in a green country environment. Studies with typically developed adults (Lee, 2015; Veit et al., 2018), and children (Wells, 2000; Dadvand et al., 2015; Schutte et al., 2017) or atypical developed subjects (Kuo and Taylor, 2004) showed a positive role of natural environments on cognition and a stress reduction after the immersion in a natural environment.

Assignment and circumstances demanding that subjects deliberately direct attention or inhibit unwanted stimuli, thoughts, or impulses utilize a shared mechanism leading to fatigue (Kaplan, 1995). After prolonged or intense use of this mechanism, fatigue is established, and an amplified difficulty to pay attention and to inhibit impulses are revealed. These findings also explain the behavior and performance of individuals without ADHD who temporarily show many of the ADHD patterns.

The ADHD symptoms and “attention fatigue” are so similar that the Attention Deficit Disorders Evaluation Scale has been used also to evaluate attention fatigue (Wells, 2000). However, differently from ADHD, attention fatigue has been measured as a transitory situation; when the deliberate attention mechanism has the occasion to rest, fatigue vanish and behavior and performance improve (Kuo and Taylor, 2004). According to Kaplan, natural environments help in the rehabilitation from attention fatigue, partially because they effortlessly enroll the mind, (Ulrich, 1981) giving a breathing space from having to voluntarily direct attention. Thus, the sense of wellness commonly experienced after spending time in a natural settings may in part reflect a systematic restorative effect on directed attention. Even a short visits in a green environment shows a positive effect on perceived stress release compared to built-up environment (Tyrvainen et al., 2014). Previous studies have reported the associations between the presence of a green space near home and significant lower stress levels (Gidlof-Gunnarsson and Ohrstrom, 2007). Even if the exact mechanism explaining how the natural environment can reduce the stress level is still unclear, it has been documented that the natural environment safeguards the negative impact of stressors (e.g., Brown et al., 2013) and decreases recovery time following exposure to a stressor (van den Berg et al., 2014).

In addition, an increase of gray matter volume in the left and right prefrontal cortex and in the left premotor cortex and an increase of white matter volume in the right prefrontal region, in the left premotor region, and in both cerebellar hemispheres has been reported in subjects exposed to a natural environment Dadvand et al. (2015). Some of these regions partly overlapped with regions related to cognitive test scores (prefrontal cortex and cerebellar and premotor white matter) and peak volumes in these regions predict higher working memory performance and decreased inattentiveness. Schutte et al. (2017) reported a positive effect of walking in natural versus walking on urban environments on attentional performance only in school-aged children but not in kindergarten-aged children. These results probably were linked to the important changes occurred in higher cognitive functions that in part reflect changes in brain structure after the age of 5 (Dennis and Thompson, 2013).

Following the numerous and now proven brain changes occurring during childhood and adolescence, with an increase in brain plasticity and therefore in the brain system’s vulnerability, the environment may play an important role in the social and cognitive development.

The effect of environment on brain activity and associated moods has been described by Ulrich (1981), who found increased alpha waves and less subjective emotional stress among participant exposed to the vision of slides of nature.

The Urban fragmentation and the reduction of urban green spaces are one of the cause of the decrease of wellness in the present time since green spaces have been reported to reduce stress and increase well-being (Tzoulas et al., 2007; Berto, 2014; Hartig et al., 2014).

Hedblom et al. (2019) describe that habitat incorporating multisensory stimuli of green areas (forest and park), are able to mitigate physiological stress induction and promote a more ready stress decrease in comparison to an habitat without green areas (i.e., urban areas).

## The Importance of Social Stimuli in Attentional Functions During Development

The ability to control social information influencing attention is important for the child’s adaptive development. Among the social signals, the faces are the most important source of social information and the control of the gaze could have a fundamental role in the development of socialization. The direction of the gaze provides a very strong signal that could be used to learn information about the internal states of other people. The peculiarity of the faces used as stimuli for the development of attention systems has been demonstrated in many studies using different methods. Frischen et al. (2007) investigated the meaning of perception of the gaze and its impact on other people, also investigating the shifting behavior of gaze direction and its influence on other persons and on joint attention in children, adults and in the clinical population. The authors focused on the paradigm of signaling the gaze direction, used to investigate the mechanism of joint attention. The contribution of this paradigm is significant and can bring advances in knowledge in different fields of psychology and neuroscience. The eyes and the surrounding region are highly expressive and can communicate complex mental states such as emotions, beliefs, and desires.

The authors focused on the aspect of gaze perception, the use of gaze direction to shift visual attention, the automatic propensity to direct attention to an object that other people are also observing.

Shared attention has been investigates in children for decades. Cognitive control is mediated by the suppression of interferences between different competing responses and is evaluated through experimental paradigms using attentional tasks such as the flanker task (Eriksen and Eriksen, 1974).

In a study of our group (Federico et al., 2013) comparing a social variant of the Attention Network Test (ANT) with schematic and real faces, with an ANT with fish -shaped stimuli in a sample of adults, we shown that the photographs of the faces positively influenced executive control in a significant way with respect to the performance of ANT participants with targets in the shape of fish or schematic faces. This evidence suggests that the participants engaged in a more effective cognitive control process during exposure to relevant social stimuli, demonstrating that people automatically focus more attention on real central faces, excluding the faces flanking the target, and achieving speed response time. This advantage was not observed for non-socially relevant stimuli. An advantage in the orienting system using social stimuli was observed also in children. We also (Federico et al., 2017) tested 5–10 years-olds children from 5 to 10 in the same three versions of the task demonstrating a larger cognitive interference (i.e., slower RTs and a higher percentage of errors to incongruent relative to congruent conditions) when fish and schematic faces were presented, compared to photographs of real faces stimuli. These issues advice that, similarly to adults, children have a higher control of social information as compared to non-social information. This is in line with several recent studies using different methods describing a higher interference effects from non -social than from eye-gaze stimuli (Barnes et al., 2007; Dichter and Belger, 2007; Kuhn and Tatler, 2011).

Many data suggested that social features are preferred over competing for physically salient objects when viewing complex naturalistic scenes (Birmingham et al., 2009; End and Gamer, 2017; Flechsenhar and Gamer, 2017; Rosier et al., 2017). In particular, End and Gamer (2017) assumed that social features in complex naturalistic scenes would be primary processed, regardless of their physical saliency. They shown that social attention and physical saliency collaborate in predicting the very first fixations during scene processing. More specifically, they reported that the preferred processing of social features in complex naturalistic scenes does not only depend on a voluntary controlled mechanism but reflects the influence of a reflexive and automatic process trading off physical saliency by the presence of social features on very early fixations. Another important aspect is the finding that social relationships may be a stress reduction facilitator factor. Social support has been widely studied as a factor that minimizes the effects on stress, and the results are somewhat striking. Sherman and colleagues demonstrated that social support is protective against depressive symptoms. In the aging brain social support was positively associated with right medial prefrontal cortical thickness whereas amygdala volume was negatively correlated with social support and positively linked to stress (Sherman et al., 2016): the authors suggested that social support was straightly related with brain circuitry that has been involved in psychological well-being. These results move ahead the understanding of how supportive relationships are significantly linked to brain circuitry implicated in emotional and social processing. During childhood, early stressful events affects people’s capacity to control, or regulate their emotions and the brain regions supporting these skills. For example, children with high stress appear to have more difficulties in containing negative emotions like anger or anxiety (Burkholder et al., 2016).

## Conclusion

Attentional control processes are multimodal and include the capacity to achieve and maintain a vigilance state (alerting system), the capacity to focus attention for a protracted period to specific, mainly, visuospatial stimuli (orienting system) as well as the capacity to inhibit challenging responses and regulate/monitor actions (executive component or conflict resolution system; Sperduti et al., 2016). Attentional processes dynamically interact with higher-order cognitive processes; therefore, the proper functioning of attention circuits is the basis of good general cognitive functioning. In particular, stress reduction is a determining factor for the optimal works of attention circuits.

**FIGURE 1:**
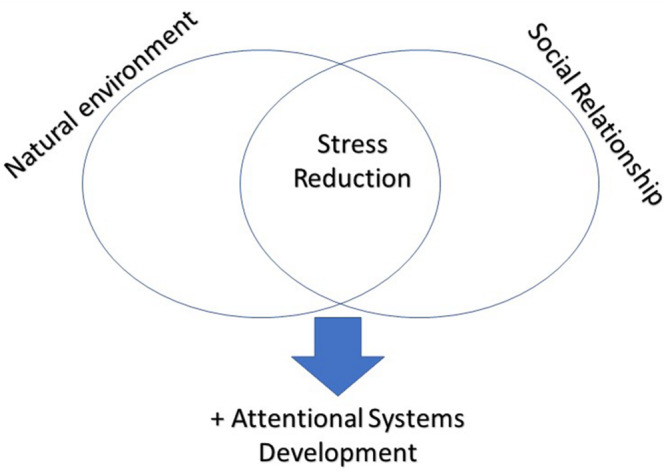
To be exposed to natural environment and to be immersed in satisfying social networks contribute together to the stress reduction, which positively affects the development of attentional systems.

In conclusion, as you can see in Figure 1, given the importance of stress reduction for the correct development of cognitive processes and in particular of attentional processes, promoting the immersion in a natural environment and the involvement on many social relationships could be the key to optimizing cognitive and attentional processes.

## Author Contributions

The author confirms being the sole contributor of this work and has approved it for publication.

## Conflict of Interest

The authors declare that the research was conducted in the absence of any commercial or financial relationships that could be construed as a potential conflict of interest.
